# A Cautionary Tale of Sexing by Methylation: Hybrid Bisulfite-Conversion Sequencing of Immunoprecipitated Methylated DNA in *Chrysemys picta* Turtles with Temperature-Dependent Sex Determination Reveals Contrasting Patterns of Somatic and Gonadal Methylation, but No Unobtrusive Sex Diagnostic

**DOI:** 10.3390/ani13010117

**Published:** 2022-12-28

**Authors:** Beatriz A. Mizoguchi, Nicole Valenzuela

**Affiliations:** Department of Ecology, Evolution and Organismal Biology, Iowa State University, Ames, IA 50011, USA

**Keywords:** epigenetic DNA methylation, temperature-dependent sex determination—TSD, bisulfite conversion of immunoprecipitated DNA MeDIP-BS-seq, methylation-sensitive ELISA, methylation-sensitive PCR, methylation-sensitive restriction enzyme, vertebrate reptilian turtle, somatic versus gonadal tissue, sexing diagnosis, conservation ecology

## Abstract

**Simple Summary:**

Identifying the sex of turtle hatchlings is important to assess the sex ratio of populations, which is important to study their ecology and evolution and for conservation programs. However, turtle hatchlings rarely display morphological differences detectable to the naked eye, and existing sexing techniques are either harmful, lethal, or non-viable for turtles with temperature-dependent sex determination. We investigated two methodologies that rely on differences in DNA methylation, a modification that occurs naturally in the DNA without changing its sequence, but that affects the expression of genes. As DNA methylation is known to differ in the gonads of male and female painted turtle hatchlings, we investigated whether the same is true in their tails We found that the painted turtle displays differential DNA methylation in the gonads, but not in the tails. We conclude that DNA methylation is tissue-specific in the painted turtle and that this epigenetic modification plays an important role in sexual development in this species but not in the somatic tissue of the tails.

**Abstract:**

**Background**: The gonads of *Chrysemys picta*, a turtle with temperature-dependent sex determination (TSD), exhibit differential DNA methylation between males and females, but whether the same is true in somatic tissues remains unknown. Such differential DNA methylation in the soma would provide a non-lethal sex diagnostic for TSD turtle hatchings who lack visually detectable sexual dimorphism when young. **Methods:** Here, we tested multiple approaches to study DNA methylation in tail clips of *Chrysemys picta* hatchlings, to identify differentially methylated candidate regions/sites that could serve as molecular sex markers To detect global differential methylation in the tails we used methylation-sensitive ELISA, and to test for differential local methylation we developed a novel hybrid method by sequencing immunoprecipitated and bisulfite converted DNA (MeDIP-BS-seq) followed by PCR validation of candidate regions/sites after digestion with a methylation-sensitive restriction enzyme. **Results:** We detected no global differences in methylation between males and females via ELISA. While we detected inter-individual variation in DNA methylation in the tails, this variation was not sexually dimorphic, in contrast with hatchling gonads. **Conclusions:** Results highlight that differential DNA methylation is tissue-specific and plays a key role in gonadal formation (primary sexual development) and maintenance post-hatching, but not in the somatic tail tissue.

## 1. Introduction

Epigenetic modifications mark DNA nucleotides chemically without altering their sequence in response to normal environmental signals (e.g., nutrition and temperature fluctuations) [1] or to environmental stressors during development, including sexual development [2]. DNA methylation is the most commonly studied epigenetic modification. It is a trait characterized by the replacement of the carbon 5′ of a deoxycytidine next to guanine (CpG) by a methyl group which alters the conformation of the major groove of the DNA, which in turn, affects the interaction of the DNA with the transcriptional machinery [3,4]. Hence, DNA methylation changes are tightly related to gene regulation [5]. As methylated cytosines undergo spontaneous deamination resulting in C to T (thymine) mutations, the abundance of CpG dinucleotides is reduced over evolutionary time from the expectation based on the frequency of Cs and Gs in the genome [6,7]. Various studies demonstrated that DNA methylation is sexually dimorphic in the developing or post-hatching gonads of vertebrates with temperature-dependent sex determination (TSD), including turtles [6,8,9,10,11] and alligator [12], and in fish with a mixed system of genotypic-sex determination susceptible to thermal effects (GSD + TE) [13,14,15]. These observations raise the possibility that DNA methylation, if it were sexually dimorphic in somatic tissues, could be used as a non-lethal sex diagnostic.

Sex diagnosis has important implications for basic and applied biology, as it is necessary to study a myriad of sexually dimorphic traits [16], as well as to monitor sex ratios to study population dynamics or to evaluate conservation efforts [17,18,19,20,21]. Sexing individuals is also important for research on sex determination in turtles to understand the effects of environmental factors (or lack thereof) on sexual development and its evolutionary consequences (e.g., [22,23,24,25,26]). As hatchling turtles usually display little sexual dimorphism that could be easily discerned by external observation, the development of sexing techniques is necessary.

Unfortunately, some earlier sexing techniques used in turtles are either lethal, as they rely on the gonadal inspection and/or gonadal tissue collection [27,28], while others require special training or equipment, such as laparoscopy/endoscopy of live animals, radioimmunoassay (RIA) of circulating hormone levels, or immunohistochemistry [29,30,31,32,33]. Non-invasive geometric morphometric techniques were also developed for a variety of species [34,35,36,37], but fast and simple field techniques remain elusive for young turtles. In recent years, alternative non-lethal sexing methods were reported, such as penile stimulation with vibrators and penis eversion by hind limb and neck stimulation [38,39], that are applicable in the field without harming the animal. Regrettably, results obtained with these last two methods are affected by the stress level of the animal post-capture and thus, are not reliable [38,39].

Molecular sexing methods were developed to identify the sex of turtles by detecting sex-specific genetic sequences or gene dosage in species with sex chromosome systems of GSD [reviewed in [40]]. For instance, gene dosage was detected with sexing primers for *Apalone spinifera*, *Glyptemys insculpta* and *Glyptemys muhlenbergii* [41], whereas quantification of the sex-specific abundance of rRNA repeats using qPCR was used to sex *A. spinifera* and other trionychid species such as *Pelodiscus sinensis* and *Chitra indica* [40,42]. In contrast, molecular sexing of turtles with temperature-dependent sex determination (TSD), who lack sex chromosomes or any consistent genotypic differences between the sexes [43], has been accomplished by measuring circulating testosterone levels after a hormonal challenge [17], and more recently, by sex-specific circulating proteins in neonate blood [44]. However, no study has explored the use of epigenetic markers for non-lethal sex diagnosis in any turtle. Any sexually dimorphic DNA methylation present in easily sampled somatic tissues (such as tail clips) could be used as a non-lethal sex diagnostic.

DNA methylation can be measured globally (genome-wide) by DNA methylation-sensitive ELISA (Enzyme-Linked Immunoassay) [45], or by high-throughput sequencing of immunoprecipitated methylated DNA (MeDIP-seq) [6]. DNA methylation can also be assessed locally (gene-by-gene or region by-region) by MeDIP-seq, by sequencing bisulfite-converted DNA (BS-seq) (which reveals the methylation status of individual nucleotides) [45], or by PCR after DNA digestion with a methylation-sensitive restriction enzyme [46]. The latter is the simplest method and was applied to sex chickens [46], a GSD species whose CpG-rich region on the Z chromosome, called MHM region (Male Hyper-Methylated region), constitutes an ideal male-specific molecular marker. This technique was also successful to identify differential methylation in the gene *Fezf2* in TSD turtle gonads [6], yet it is unknown if somatic tissues display the same pattern.

Here, we investigated the global and local DNA methylation in a somatic tissue (the tail) of *Chrysemys picta* hatchlings using a multi-pronged approach, to test the hypothesis that differential DNA methylation exists in somatic tissue and can be used as a sexing technique for TSD turtles. We chose tails because their shape is sexually dimorphic in turtles of the family Emydidae to which *C. picta* belongs [47], and because tail clips can be easily collected in the field without sacrificing the individual. Our analysis included methylation-sensitive ELISA (global), plus a novel hybrid method we developed to provide global and local methylation information by combining MeDIP-seq with BS-seq (MeDIP-BS-seq). Our novel MeDIP-BS-seq offers an alternative to quantify methylation in genes or regions while also providing base-by-base methylation information simultaneously. Additionally, we assembled the methylomes of tails of male and female *C. picta* hatchlings to identify candidate molecular sex markers in TSD turtles for PCR detection after methylation-sensitive DNA digestion.

## 2. Materials and Methods

### 2.1. Tissue Collection and DNA Extraction

Freshly laid eggs were collected from an Iowa turtle farm and transported in moist vermiculite to the laboratory for incubation following standard protocols [48]. Specifically, eggs were cleaned from excess mud, marked with a unique ID, randomly assigned to boxes with moist sand (30 eggs per box), and placed in incubators at 26 °C (Male Producing Temperature—MPT), 28 °C (Pivotal Temperature—PivT, which produces males and females in equal numbers) and 31 °C (Female Producing Temperature—FPT). Boxes were rotated daily in a clockwise fashion to control for potential temperature gradients within the incubators. Moisture inside the egg boxes was maintained constant by replacing evaporated water weekly. We obtained 20 hatchlings from the 26 °C treatment, 20 hatchlings from the 28 °C treatment and 23 hatchlings from the 31 °C treatment. Hatchlings were assigned a unique ID according to their incubation treatment and order of hatching and were notched at their carapace scutes for identification [49]. Hatchlings were raised for 3 months in water tanks at 26 °C, fed ad libitum and cleaned daily. All animals were euthanized by a lethal injection of propofol and sex was determined by visual gonadal inspection. We collected tail clips from each hatchling and preserved the tissue in RNA later at −20 °C until further processing. All procedures were approved by Iowa State University IACUC.

DNA was extracted separately from the tail of each individual collected as described above, using Gentra Puregene DNA extraction kits (Gentra), and following the manufacturer’s instructions. DNA quality and quantity were assessed by Nanodrop Spectrophotometer and 1% agarose gel electrophoresis. DNA was diluted to 200 ng/uL and stored at −20 °C until processing.

### 2.2. Global Methylation Analysis via ELISA

We measured global methylation levels in 20 randomly selected individuals per temperature (26 °C, 28 °C and 31 °C) via methylation-specific ELISA using the MethylFlashTM Global DNA Methylation (5-mC) ELISA Easy Kit (Epigentek), following the manufacturer’s instructions. DNA samples were diluted to 10 ng/uL for ELISA. Reactions were run in a Chromate 4300 machine at the Iowa State University proteomics facility. All plates included a positive control standard curve from the kit plus a turtle-specific standard curve of eight standards obtained by serially diluting (1:1) a sample of pooled DNA from all individuals.

The normality of the absorbance values was tested using QQ plots in RRPP [50], and results indicated that no data transformation was necessary (Supplementary Figure S1). We converted the absorbance values to methylation percentage, following the equation [51]:

(1) $$5\mathrm{mC}\%=\frac{\mathrm{SampleOD}-\mathrm{NCOD}}{\mathrm{Slope}\;\times \;S}\times 100\%$$

where 5 mC% = percentage of 5-methylcytosines, OD = optimal density, NC = negative control, Slope = standard curve slope, S = input DNA in ng. For the statistical analysis of these values, we evaluated first if the standard curves of positive control (provided with the kit) and our turtle standard curves were linear using a generalized linear model (GLM). Then, we performed an ANCOVA to compare slopes between these two types of standard curves. Next, we tested for differences in global methylation using ANOVA. Tests were applied first to the calculated methylated percentages that represent the total 5-mC fraction in the sample accounting for the kit’s specificity in detecting DNA methylation, given that it is calculated proportionally to the OD intensity measured [51]. Second, ANOVA was applied to the absorbance values, following a traditional ELISA data analysis [52]. As the interaction between temperature and sex was not significant for the analysis of 5 mC% in the full factorial ANOVA (*p* > 0.05), we performed a reduced ANOVA that excluded the interaction term. Additionally, because the temperature and sex terms were not significant in the reduced model, we then tested for differences combining samples by sex (26 °C male + 28 °C male and 31 °C female + 28 °C female), and by temperature (31 °C, 26 °C and 28 °C). On the other hand, because the sex and temperature interaction was significant for the absorbance values (*p* < 0.05) we did a pairwise comparison of all temperature by sex combinations.

### 2.3. MeDIP-BS-Seq Library Construction and Sequencing

Twenty random samples of DNA per incubation treatment (26 °C and 31 °C) were divided into two groups to obtain two biological replicates of pooled DNA per temperature (10 samples per pool). DNA was processed by EpiGentek using a hybrid approach we developed to detect methylated regions. Specifically, methylated DNA was immunoprecipitated first (5 mC MeDIP) and then subjected to bisulfite conversion, after which Illumina NextSeq 500 libraries were prepared and sequenced at Duke University sequencing facility (75 bp PE sequencing). This hybrid sequencing approach was designed to quantify methylation via MeDIP, which targets mostly CpG-rich regions and to assess the base-by-base cytosine methylation status of the immunoprecipitated DNA from the bisulfite-conversion.

### 2.4. Methylome Assembly and Analysis

The quality of the library reads was assessed by FASTQC [53] followed by an adaptor trimming step using trimgalore [54]. Trimmed reads were quality controlled in an additional step via FASTQC to check for the adaptor removal. We used the *Chrysemys picta* 3.0.3 genome assembly [55] as a reference to map the MeDIP-seq+ BS-seq reads using Bismark [56]. We applied the genome preparation step, by which the software converts the reference genome into a 3-base genome (cytosines are converted to thymines and adenines are converted to guanines), followed by single- and non-directional read mapping, with a score of −120. Alignments were sorted using Samtools [57], and then imported into RStudio [58] using the process BismarkAln from methylkit [59] for further analysis. The conversion rate was calculated by methylkit as the number of thymines divided by coverage for each non-CpG cytosine. Non-methylated cytosines are converted to uracils during the bisulfite conversion, which in turn are converted to thymines during PCR amplification. Coverage is calculated by the number of reads per base, with a minimum of 10 reads per base to ensure the high quality of the data and methylation percentage [59].

We tiled the genome in windows of 1000 bp for differential methylation analysis, as recommended by methylkit. This tiling process allows methylkit to summarize methylation information using these windows rather than individual bases. Following the window-tiling process, we calculated differential methylation using the methylkit function “calculateDiffMeth”, with the q-value set at 0.01. We used Bedtools [60] to obtain region coordinates of exons, introns, intergenic regions and promoter regions (i.e., 500 bp, 1000 bp and 3500 bp upstream of exon 1) from the *C. picta* genome ver 3.0.3 [55] from NCBI. Regions with *p* < 0.05 were annotated using genomation [61] to identify methylation present in promoters, introns, exons and intergenic regions. Differential methylation analysis was also performed at individual nucleotides, using the same functions of methylkit and q-values as for the analysis by windows.

In an alternative approach, we run a coverage-based analysis on edgeR [62], to identify differentially methylated regions using 500 bp windows, following [6]. For this, we created a count table (Data S1) from our alignments, using “bedtools coverage” and imported it into RStudio for edgeR analysis. We used the quasi-likelihood F-test (QLF) to calculate differential methylation, and methylation levels were measured as the natural log of the Counts Per Million (logCPM) [63]. Scripts used in this study can be found in Material S1.

### 2.5. DNA Digestion and Methylome Validation by PCR

**Figure 1:**
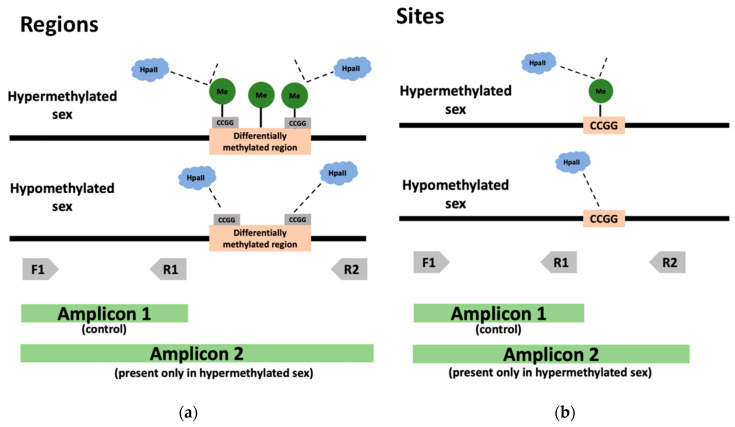
Methylation-sensitive PCR to validate the candidate regions and sites for tail sexing. In regions (**a**), the hypermethylated sex has one or more restriction cleavage sites for the methylation-sensitive restriction enzyme HpaII (CCGG) surrounded by methyl groups. In candidate sites (**b**), a single differentially methylated cytosine is located within the restriction cleavage site. In both these cases (regions and sites), methylation within the restriction sequence prevents DNA digestion by HpaII, while hypomethylation at this site permits DNA digestion. Primers F1 and R1 produce an amplicon irrespective of DNA methylation at the candidate region or site, and thus serve as a control, whereas amplicon 2 is only produced by primers F1 and R2 in the presence of methylation at the restriction site (when DNA digestion is prevented).

Twenty regions that showed significant differential methylation (*p* < 0.05) between males and females in the previous analyses were selected as candidate regions for a role in sex diagnostics and were inspected visually in Geneious [64]. Regions were selected for validation according to the difference in methylation between treatments (26 °C and 31 °C), and to the presence of the restriction site (CCGG) recognized by the methylation-sensitive restriction enzyme HpaII. To validate regions, we designed primers (Table S1) according to the location of the highest coverage peak within the region (i.e., the peak location enriched with aligned reads which indicates a reliable methylation call) and the location of the restriction site. To validate methylation at specific bases, we designed primers according to the differential methylated site location, given that it is the only area where there is a difference in methylation that would be detected by the restriction enzyme (Figure 1).

DNA (100 ng per reaction) was digested with HpaII (Thermo Fisher, Waltham, MA USA), following the manufacturer’s instructions, and digestion was verified visually by comparing digested and undigested DNA in 1% agarose gels stained with ethidium bromide (EtBr) against a 1 kb plus ladder (Invitrogen). Undigested DNA should concentrate above the 25 kb standard whereas digested DNA (unmethylated) produces a smear in the gel between 1.5 Kb and 12 kb. PCR amplification used 10 ng of digested and undigested DNA (control) in 15 µL reactions containing 1× Tag buffer, 1.5 mM MgCl_2_, 0.2 mM dNTPs, 0.4 U Taq polymerase, 10.5 µL water, and a 0.4 µM primer cocktail containing the three primers in equimolar concentrations. PCR conditions included an initial denaturing step at 94 °C for 3 min, followed by 35 cycles of denaturing at 94 °C for 30 s, annealing at 58 °C for 30 s, and extension at 72 °C for 90 s. Amplicons were visualized in EtBr-stained 1% agarose gel, and their size estimated against a 1 kb plus ladder (Invitrogen, Waltham, MA USA).

## 3. Results

### 3.1. Global Methylation by ELISA

**Figure 2:**
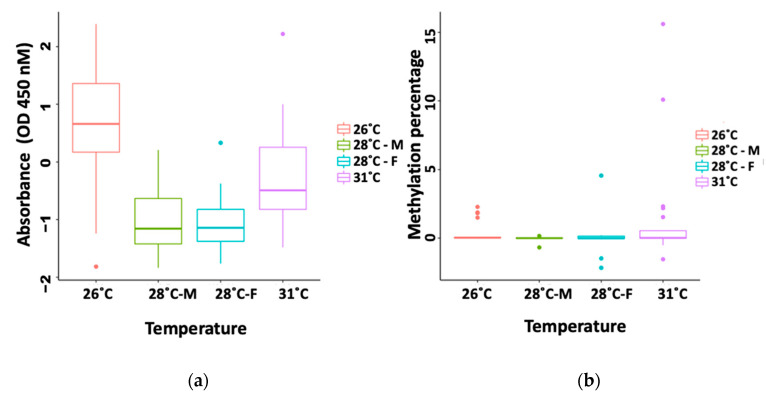
(**a**) Absorbance and (**b**) percent 5-mC DNA methylation values by temperature and sex measured by ELISA in the tail of *Chrysemys picta* hatchling tail tissue.

In order to investigate whether overall differential methylation in *C. picta* tails is present such that it would be a good indicator of the individual’s sex, we performed an ELISA assay to detect global methylation differences between temperature treatments (26 °C, 28 °C, and 31 °C). Standard curves were linear and displayed an R^2^ = 0.95 and R^2^ = 0.99 for the kit positive control (PC) and the turtle standard curve (TC), respectively. An ANCOVA revealed no significant difference between the slopes of the PC and TC standard curves (*p* = 0.6). Our ANOVA analysis between temperature and sex groups uncovered no significant difference in global methylation percentage or absorbance between males and females that could be used as a sexing marker (*p* > 0.05) (Figure 2, Table 1), although a permutation procedure [50] detected significantly higher within-group variance in percent methylation of 31 °C females than 26 °C males (but not among other groups).

### 3.2. MeDIP-BS-Seq Methylome Assembly and Analysis

A reference methylome was assembled using pooled reads from the 26 °C and 31 °C temperature treatments, which in *Chrysemys picta* (TSD) produce exclusively males and females, respectively. All reverse reads (R2) from the paired-end RNA-sequencing were comprised of guanines (Gs), an artifact later found to be commonly caused by the two-color Illumina Nextseq chemistry which over-calls no-signal N bases as high confidence Gs. Therefore, reverse reads were discarded from further analysis, and the methylome assembly and analysis were based on single (forward) reads only, which are typically used for bisulfite sequencing [65,66,67]. We obtained a mapping efficiency of reads to the CPI 3.0.3 genome between 60% and 70% and a bisulfite conversion rate for all samples between 83–92% (83.15% and 90.41% for females, and 91.56%, and 91.81% for males). Similar to the ELISA results, Bismark detected no differences between the sexes in global methylation levels for cytosines in CpG context (78.60% and 84.00% for female, and 81.00% and 81.30% in males). In contrast, cytosines in non-CpG context (CHH) exhibited lower methylation in females than in males in these CpG-rich regions pulled down during MeDIP (4.80% and 4.30% for females, but 6.90% and 8.60% for males) despite female samples experiencing lower conversion efficiency which would have caused the overestimation of their methylation level compared to males. However, having duplicates does not provide enough power to detect the significance of this difference using a *t*-test (*p* > 0.05).

At the 1000 bp window level (using methylkit), we detected 164 differentially methylated regions while at the 500 bp window level (using edgeR) we identified 761,800 differentially methylated regions. No region exhibited a presence/absence pattern, that is, showing 100% methylation at one temperature (26 °C or 31 °C) and 0% methylated in the other temperature, i.e., the ideal scenario for primer design and DNA digestion by methylation-sensitive restriction enzymes, perhaps due to the lower conversion efficiency. Therefore, we selected the top three regions (Table 2) that displayed the greatest differences in fold-change of methylation levels between the 26 °C and 31 °C treatments for downstream methylation-sensitive PCR. At the site-by-site level, we detected 34 individual sites that exhibited the 100–0% methylation pattern between temperatures. Of those 34 sites, only two were located at a restriction site that would be amenable for methylation-sensitive PCR, and both were selected for further tests (Table 2).

### 3.3. Methylation-Sensitive PCR

We used DNA from 10 individuals incubated at 26 °C and 10 at 31 °C, sexed by gonadal inspection, to test for differential methylation by the PCR assay in three candidate regions and two candidate sites (Table 1). Amplification patterns did not differ between the sexes. Indeed, amplicons of the size expected if methylation was absent were observed between undigested and digested samples in both sexes. Visual inspection of the DNA template before PCR in an agarose gel showed a smear for the digested DNA sample but not for the undigested DNA. Thus, the PCR results revealed that the restriction enzyme did not digest the DNA at the candidate regions at a significant level in either sex. Therefore, the regions selected because they exhibited the greatest differential methylation in the methylome analysis exhibited negligible methylation in both sexes when tested by PCR, and thus, cannot be used as a sex diagnostic.

## 4. Discussion

### 4.1. A Novel Complexity-Reduction and Site-by-Site Approach for DNA Methylation Analysis

DNA methylation is an important biological process, and several methods have been designed to discern DNA methylation patterns in ecological, evolutionary and medical research [68,69,70]. In this study, we tested a new approach to study DNA methylation, by combining MeDIP and BS-Seq. We applied this new hybrid technique to study the DNA methylation of somatic tissue (tail clips) of *C. picta* hatchlings with the goal of identifying molecular markers that could serve as a sex diagnostic tool. To our knowledge, this is the first time a MeDIP + BS-Seq hybrid approach has been used in a DNA methylation study. This complexity reduction procedure allowed us to enrich the genomic DNA samples to those regions with higher DNA methylation via MeDIP, and to obtain information on the individual base methylation status from the BS-Seq. Other methods used to study CpG density at one or a few regions of interest, generally combine restriction enzymes and fluorescence along with bisulfite conversion and sequencing. These methods include either tagging methylated CpG dinucleotides, or labeling S-adenosylmethionine—(SAM—a methyl donor) to incorporate methyl groups to bisulfite converted PCR amplicons, in order to identify all the CpGs that are methylated in a fragment [69,71,72]. These methods focus on exploring regional CpG density in targeted genomic areas, while our approach allows an unbiased genome-wide DNA methylation profiling [73]. Furthermore, these other methods have limited data acquisition compared to our hybrid method, as they are highly dependent on the quantity and location of the restriction sites, on the quality of the digested DNA, and because they are also restricted to genomic regions of known sequence [69,73]. In contrast, our hybrid method is able to capture genome-wide regions that are enriched in methylation through the MeDIP-Seq step and to provide additional site-specific information thanks to the bisulfite-sequencing step. We achieved satisfactory mapping results (60–70%) using Bismark compared to the 56% and 77% mapping efficiency attained for human blood samples [65,74], and 24.6% using Bismark on mice [75]. This was true despite the fact that our mapping efficiency was reduced compared to other studies of DNA methylation in turtles that used exclusively MeDIP [6], partly because we used single-end reads, and partly because no software exists specifically designed to handle the combined MeDIP + bisulfite data. In addition, we note that the calculated bisulfite conversion efficiency obtained in this study (83–92%) is lower compared to what others have obtained (99%) [76]. This is expected to increase false positives, as some unmethylated cytosines were not converted to uracils and would have been counted as methylated cytosines during the analysis (i.e., misinterpreted as being protected from deamination by the presence of a methylation mark). Thus, subtle but biologically significant differential methylation between the sexes may have been obscured (particularly higher male than female methylation, since conversion efficiency was lower in female than male tail samples).

Further optimization of the MeDIP-BS-seq protocol is warranted as well as improvements in bioinformatics pipelines to handle hybrid data of this kind to improve results, and we hope that our work will foster new developments in this area. Importantly, because our novel hybrid method was successful in providing a genome-wide assessment of the DNA methylation status in hatchling tails and thus, it should be applicable at a broad taxonomic scale to other DNA methylation analyses of somatic or gonadal tissue, and particularly useful to reduce the complexity of samples for the study of large genomes.

### 4.2. Inter-Individual Variation in DNA Methylation Exists in Both Males and Females

Despite identifying multiple differentially methylated regions and sites in the tails among individuals, and overall higher CHH methylation in females than males in the regions pulled down by MeDIP (which are CpG-enriched), no reliable sexing marker was detected by methylation-sensitive PCR using any of the top candidate regions. Indeed, although differential methylation was identified with the MeDIP-BS-Seq data between pooled samples of males and females, the differences turned out to not be dichotomous enough for the restriction enzyme to yield sexually dimorphic methylation-sensitive PCR markers. Further, the difference in overall CHH methylation levels detected here does not provide a cheap diagnostic tool for sex TSD individuals at the scale needed for population-level analysis. Thus, the current method for sexing TSD turtles with relative reliability includes the recent immunoassay of circulating AMH (Anti-Mullerian Hormone) protein in the blood, which was tested in two species (*Trachemys scripta* and *Caretta caretta*) and was 100% accurate in neonates, although it is less accurate at an older age (accuracy dropped to 90% in 2.7–6 mo old juveniles) [44] and is expected to drop in accuracy once the Mullerian ducts are fully resorbed in males. While sampling blood is minimally invasive (albeit not always easy), this immunoassay is simpler than the radioimmunoassay of circulating testosterone after the FSH challenge, which has been applied to sea and freshwater turtle hatchlings [29,30,77]. Importantly, the only non-invasive sexing method to date is able to discern very subtle external sexual dimorphism using landmark-based geometric morphometrics, which was 90%-98% accurate to diagnose the sex of *Podocnemis expansa* and *C. picta* hatchlings [24,34], and was applied to sex hatchlings of *Podocnemis lewyana* and *Chelydra serpentina* [22,78].

### 4.3. Contrasting Patterns of DNA Methylation between Somatic and Gonadal Tissues

Our results concur with earlier reports of extensive methylation in the genome of *C. picta* hatchlings [6], yet, the response of DNA methylation to incubation temperature differ drastically between gonadal and somatic tissue (i.e., tails). Indeed, previous studies uncovered sexually dimorphic DNA methylation in the gonadal tissue of TSD turtles (*C. picta* and *Lepidochelys olivacea*) [6,10], whereas we observed mostly monomorphic DNA methylation in tails. This monomorphism was evident both at the global (genome-wide, determined by ELISA) and local (at small regions and at individual sites, determined by MeDIP-BS-seq) levels. Gonads are sexually dimorphic tissues by definition, and in *C. picta*, they display differential DNA methylation in hatchlings and differential gene expression patterns since early development [6,79,80,81,82,83,84]. Turtle tails exhibit morphological differences between the sexes that are relevant for mating, such as contrasting size, texture, or the relative position of the cloaca [85]. For instance, tails are sexually dimorphic in *Trachemys scripta* turtles, an emydid close relative of *C. picta* [47,86], yet we did not observe sexually dimorphic DNA methylation in the hatchling tails, perhaps because the tail dimorphism has not yet developed in painted turtle hatchlings [47] or because its development is not controlled epigenetically via DNA methylation. Further, the painted turtle does not rely on male combat or forced insemination, two mating strategies linked to male-specific body size and shape [85,86]. On the contrary, *C. picta* mating relies on female choice where pre-coital male behavior and display structures such as foreclaws and coloration are relevant [85]. Nonetheless, the discrepancy between our results and those previously reported for gonadal tissue underscores the importance of DNA methylation for the sex-specific maintenance and/or function of the gonads but not for some somatic tissues, such as the tail. Future studies should explore the sexual dimorphism of DNA methylation in other somatic tissues such as blood in turtles, which can be collected non-lethally. Blood exhibits sexually dimorphic DNA methylation in humans linked to other traits [87] and serves as a biomarker for multiple medical purposes, such as detecting aging [88] or cancer types [68,89,90].

## 5. Conclusions

In conclusion, here we build the somatic methylome of a TSD turtle with the goal to identify molecular sex markers. We found substantial differences in tail methylation among individuals, but no consistent sex-specific pattern that could be used to diagnose the sex of hatchlings accurately using this somatic tissue, in contrast with the sexually dimorphic gonadal methylation previously reported [6]. Our results underscore the importance of DNA methylation in primary sexual development and gonadal maintenance post-hatching and highlight that sexually dimorphic methylation is not ubiquitous in the soma. Our study led to the development of a new hybrid method that combines MeDIP-Seq and bisulfite-sequencing which provides greater insight to profile the genome-wide methylation status of large genomes with relative ease, and thus should be widely applicable, but whose further optimization is warranted.

## Figures and Tables

**Table 1:** ANOVA results for absorbance and percent 5-mC DNA methylation values measured by ELISA in the tail of *Chrysemys picta* hatchling tail tissue.

ANOVA—Methylation Percentage (Full Factorial Model)				
	**Sum of Squares**	**Mean Square**	**F**	***p***-**Value**
Temperature	14.4	14.376	2.62	0.138
Sex	0.0	0.015	0.002	0.961
Temperature:Sex	8.4	8.39	1.320	0.255
Residuals	356	6.357		
**ANOVA—methylation percentage (reduced model)**				
	**Sum of Squares**	**Mean Square**	**F**	***p*** **-value**
Temperature	14.4	14.376	2.249	0.139
Sex	0.0	0.015	0.002	0.961
Residuals	364.4	6.393		
**ANOVA—methylation percentage by sex**				
	**Sum of Squares**	**Mean Square**	**F**	***p*** **-value**
Sex	9.5	9.511	1.494	0.227
Residuals	369.3	6.367		
**ANOVA—methylation percentage by temperature**				
	**Sum of Squares**	**Mean Square**	**F**	***p*** **-value**
Temperature	14.4	14.376	2.288	0.136
Residuals	364.4	6.283		
**ANOVA—absorbance (full factorial model)**				
	**Sum of Squares**	**Mean Square**	**F**	***p*** **-value**
Temperature	4.26	4.262	5.509	0.5942
Sex	0.22	0.222	0.287	**2.33e−0.6**
Temperature:Sex	21.41	21.414	27.679	
Residuals	43.33	0.774		
**Pairwise comparisons of absorbance. Mean absorbance per temperature by sex combination (diagonal, underlined), mean squares (below diagonal, italics), *p*-values (above diagonal, significant values are denoted in bold)**				
	26 °C—Male	28 °C—Male	31 °C—Female	28 °C—Female
26 °C—Male	0.5999	**0.0008**	0.126	**0.005**
28 °C—Male	*32.56*	−1.0218	**0.035**	0.771
31 °C—Female	*7.507*	*10.722*	−0.2172	**0.067**
28 °C—Female	*24.402*	*0.084*	*8.803*	−0.9854

**Table 2:** Differentially methylated 500 bp regions and differentially methylated cytosines in the tail of *Chrysemys picta* selected for methylation-sensitive PCR. Intergenic regions are located outside any specific gene.

Regions			
**CPI 3.0.3. Scaffold**	**Start Position**	**End Position**	**Gene**
NW_007359905.1	3,604,500	3,605,000	Intergenic
NC_024218.1	55,635,611	55,636,011	Intergenic
NW_007281443.1	1,473,502	1,474,001	FAM170B
**Sites**			
**CPI 3.0.3. Scaffold**	**Position**		**Gene**
NC_024218.1	18,209,143		CASQ2
NC_024220.1	23,784,593		FOSL2

## Data Availability

All files are available in Genbank BioProject accession number PRJNA681606.

## Supplementary Materials

The following supporting information can be downloaded at: https://www.mdpi.com/article/10.3390/ani13010117/s1, Data S1: Count tables produced as input to edgeR analysis; Material S1: Scripts used in differential methylation analysis; Table S1: Primer sequences used for PCR assay.
