# Supplementary material for: A Cautionary Tale of Sexing by Methylation: Hybrid Bisulfite-Conversion Sequencing of Immunoprecipitated Methylated DNA in Chrysemys picta Turtles with Temperature-Dependent Sex Determination Reveals Contrasting Patterns of Somatic and Gonadal Methylation, but No Unobtrusive Sex Diagnostic
Source: Animals (Basel). 2022 Dec 28;13(1):117. doi: 10.3390/ani13010117 (PMC9817949; doi:10.3390/ani13010117)
Supplement: Supplementary file 1 [file animals-13-00117-s001.zip › animals-2114407-supplementary.pdf]

Supplementary Materials for:

“A cautionary tale of sexing by methylation: Hybrid bisulfite-conversion sequencing of immunoprecipitated methylated DNA in Chrysemys picta turtles with temperature-dependent sex determination reveals contrasting patterns of somatic and gonadal methylation, but no unobtrusive sex diagnostic”

Beatriz Mizoguchi* and Nicole Valenzuela

Department of Ecology, Evolution and Organismal Biology, Iowa State University, Ames, IA, USA

* Correspondence: biakemi@iastate.edu

This file contains:

Table S1 – Primers used in the PCR assay

Material S1 - Scripts used in the study

Data S1 is available in a separate Data S1.txt file.

Table S1 – Primers used in the PCR assay

Regions:

NC_024218.1- F1: ATTCTCCACGGGTCTCGT

NC_024218.1- F2: CGTGGGTTCTTCTCCCA

NC_024218.1- R: GAGGATCCGTCACGCTC

NW_007281443.1- F1: GCTTCACATGCTGGGTC

NW_007281443.1- F2: CAAAACTTTCTCCTCCGCT

NW_007281443.1- R: TTGGAGCCATTAGGAAGC

NW_007359905.1 - F1: GAGTCACTGCCGCCTGTC

NW_007359905.1 - F2: GCGAGATTGATCTGTTCCCC

NW_007359905.1 - R1: TGTAGTGCCACCAGCGGT

Sites:

CASQ2 – F1: ACCAGCACAGACAACAGA

CASQ2 – F2: AGGCCACCGAAAAACAC

CASQ2 - R: TGTGTTTCTGCTCTTTCG

FOSL2 – F1: AGCAAACGCAACAGACC

FOSL2 – F2: AAACCACAAACCCCCAC

FOSL2 - R: CTTTGGTCGTGTCGGTT

Material S1 - Scripts used in the study

Read Mapping

```bash
# genome index
bismark_genome_preparation /path/to/(unconverted)genome/folder

# mapping
bismark --bowtie2 -N 0 --non_directional --score_min L,0,-0.8 /path/to/genome/index/folder/CPI-genome --se /path/to/trimmed/reads/trimmed_reads.fq
```

Methylkit analysis (in RStudio)

```R
if (!requireNamespace("BiocManager", quietly = TRUE))
  install.packages("BiocManager")
install_github("al2na/methylKit", build_vignettes=FALSE,
	repos=BiocManager::repositories(),
	dependencies=TRUE)

library(methylKit)

setwd("~/Desktop/Tail-methylome")

# Import files to R (input files are cytosine reports from Bismark sorted alignments)
file.list=list(system.file("~/Desktop/Tail-methylome","CPI-tail-F1.sam.gz"),
	system.file("~/Desktop/Tail-methylome","CPI-tail-F2.sam.gz"),
	system.file("~/Desktop/Tail-methylome","CPI-tail-M1.sam.gz"),
```

```
        system.file("~/Desktop/Tail-methylome","CPI-tail-M2.sam.gz"))


objs=processBismarkAln(location=list("CPI-tail-F1.sam.gz",
                        "CPI-tail-F2.sam.gz",
                        "CPI-tail-M1.sam.gz",
                        "CPI-tail-M2.sam.gz"),
            sample.id=list("F1","F2","M1","M2"),
            assembly="CPI303",
            treatment=c(1,1,0,0),
            save.context=c("CpG"),
            read.context="CpG",
            save.folder=NULL)

# Filter samples based on coverage
filtered.objs=filterByCoverage(objs,lo.count=10,lo.perc=NULL,
                  hi.count=NULL,hi.perc=99.9)
# normalize coverage
normalized.objs=normalizeCoverage(filtered.objs,method="median")

# Merging samples
meth=unite(normalized.objs, destrand=TRUE)

# determine C counts in 500bp window sizes
tiled.methyldb=tileMethylCounts(object=normalized.objs,win.size=500,step.size=500)
meth2=unite(tiled.methyldb, destrand = TRUE)

# differential methylation by region
myDiff=calculateDiffMeth(meth2)
```

```
# get all differentially methylated regions
myDiff95p=getMethylDiff(myDiff,difference=95,qvalue=0.01)

# annotate results
BiocManager::install("genomation")
library(genomation)

# convert methylRawList objects into GRanges
my.gr=as(myDiff,"GRanges") #regions
head(my.gr)
my.gr

# export GRanges to CSV file
regions_df = as(my.gr, "data.frame")
write.table(x = regions_df, sep="\t", file = "CPI_tail_regions_diff.csv")

#annotations need to be a bed file with 12 columns!
#convert gff to gtf (terminal)
  #module load cufflinks/2.2.1-63gfrmo
  #gffread -E CPI_RefSeq_v303.gff -T -o CPI_annotation.gtf
#convert gtf to bed12
  #module load python/2.7.14-h73plf5
  #module load py-numpy/1.13.3-py2-sx2jhbh
  #gff_to_bed.py CPI_annotation.gtf > CPI_annotation.bed

bed.file = file.path("~/Desktop/methylkit", "CPI_annotation.bed")
head(bed.file)

gene.obj = readTranscriptFeatures(bed.file, remove.unusual=FALSE)
```

```
regions.ann = annotateWithGeneParts(as(myDiff25p,"GRanges"),gene.obj) #regions

# count reads for each genetic parts
promoters=regionCounts(objs,gene.obj$promoters)
head(promoters)
introns=regionCounts(objs,gene.obj$introns)
head(introns)
exons=regionCounts(objs,gene.obj$exons)
head(exons)

# annotate differentialy methylation regions/bases
diffAnn.regions=annotateWithGeneParts(as(myDiff25p,"GRanges"),gene.obj) #regions

# annotate hypermethylated regions/bases
diffAnnhyper.regions=annotateWithGeneParts(as(myDiff25p.hyper,"GRanges"),gene.obj) #regions

# annotate hypomethylated regions/bases
diffAnnhypo.regions=annotateWithGeneParts(as(myDiff25p.hypo,"GRanges"),gene.obj) #regions

# plot the distribution o methylation across gene parts
plotTargetAnnotation(diffAnn.regions,precedence=TRUE,
        main="differential methylation annotation") #regions

# get percentage of intron/exon/promoters that overlap with differentially methylated bases
getFeatsWithTargetsStats(diffAnn.regions,percentage=TRUE) #regions


# Get Differentially Methylated Regions/Bases Based On Cutoffs
my.diffMeth=calculateDiffMeth(meth2,slim=TRUE,weighted.mean=TRUE,num.cores=1)
```

all.diff=getMethylDiff(my.diffMeth,difference=25,qvalue=0.01,type="all")

```

### edgeR analysis

##### Count table production

``` bash
# Convert SAM file to BAM
samtools view -S -b CPI-tail-F1-trimgalore_bismark_bt2.sam > CPI-tail-F1.bam
samtools view -S -b CPI-tail-F2-trimgalore_bismark_bt2.sam > CPI-tail-F2.bam
samtools view -S -b CPI-tail-M1-trimgalore_bismark_bt2.sam > CPI-tail-M1.bam
samtools view -S -b CPI-tail-M2-trimgalore_bismark_bt2.sam > CPI-tail-M2.bam

# Sort files
samtools sort file.bam > file-sorted.bam

# Create genome windows
samtools faidx CPI-genome-BSconverted2.fna
cut -f 1,2 CPI-genome-BSconverted2.fna.fai > chrom.sizes
bedtools makewindows -g chrom.sizes -w 500 > coverage-windows-500bp.bed
sed ’s/\.1/_1/g’ coverage-windows-500bp.bed> coverage-windows-500bp-2.bed

# Create coverage files for each file
bedtools coverage -a coverage-windows-500bp-2.bed -b T26-Tail-Rep1-sort.bam > T26-Tail-Rep1-
coverage.bed

# Process files to keep only columns of interest

cut -f1,2,3,4 T26-Tail-Rep1-coverage.bed > T26-Tail-Rep1-coverage-cut.bed

#Repeat to all replicates

# Pick the first male file as the lead for your count table. Then, run the following code on the other bam files

cut -f4 T26-Tail-Rep2-coverage-cut.bed > T26-Tail-Rep2-counts-only.bed

cut -f4 T31-Tail-Rep1-coverage-cut.bed > T31-Tail-Rep1-counts-only.bed

cut -f4 T31-Tail-Rep2-coverage-cut.bed > T31-Tail-Rep2-counts-only.bed

# Create the count table

paste -d' ' T26-Tail-Rep1-coverage-cut.bed T26-Tail-Rep2-counts-only.bed T31-Tail-Rep1-counts-only.bed T31-Tail-Rep2-counts-only.bed > count_table_v1.txt

# Process count table to edgeR input

cut -f1,2,3 count_table_v1.txt > count_table_chr_names.txt

cut -f4,5,6,7 count_table_v1.txt > count_table_counts.txt

sed 's/\t/:/g' count_table_chr_names.txt > count_table_chr_names_2.txt

paste -d' ' count_table_chr_names_2.txt count_table_counts.txt > count-table-v2.txt

```

```R

# Run edgeR in R

library(edgeR)

group <- factor(c(1,1,2,2))

y <- DGEList(counts=count.table.v2,group=group)

y <- calcNormFactors(y)

design <- model.matrix(~group)

y <- estimateDisp(y,design)

```
fit <- glmQLFit(y,design)

qlf <- glmQLFTest(fit,coef=2)

tab <- topTags(qlf,n=Inf)

write.table(x = tab, file = "CPI-tail_final.csv")
```
